# A Result of National Insurance

**Published:** 1913-04-05

**Authors:** 


### FROM POOR-LAW INFIRMARIES TO PUBLIC HOSPITALS.

### A Result of National Insurance.

At the present time, when circumstances point to a rearrangement and reorganisation of the whole of the British hospital system, it may be useful in this Special Number dealing with Poor-Law matters to bring out clearly the legal position of the Poor-Law infirmary in London. The position throughout England and Wales could be readily approximated, for the purposes of such a reconstruction of our hospital system, to that advocated for the Metropolis. It would be an enormous gain to everybody concerned if the Local Government Board were to follow up its action in regard to casuals by applying it to every other branch of Poor-Law work which may be equally well consolidated with equally satisfactory results. Now that the hospital question is becoming increasingly pressing every day it is well to bear in mind that there are no fewer than thirty-four Poor-Law infirmaries in Greater London, each under a separate Board of Guardians, a most wasteful and unsatisfactory system, and when one such infirmary may be crowded with patients another may be only partially filled.

### Beds for Insured Persons.

The actual position, so far as the latest figures in our possession are concerned, is that the whole thirty-four infirmaries contained upwards of 19,000 beds, of which less than 17,000 were daily occupied on an average. At the present time no Insurance Committee is at liberty to make arrangements to send insured persons as in-patients to any of these infirmaries, because they are managed by the Poor-Law authorities, and section 16 (i) of the National Insurance Act contains a proviso preventing Insurance Committees making arrangements with Poor-Law authorities. It will, therefore, be seen that until these infirmaries are taken out of the hands of the Poor-Law authorities a large number of hospital beds which might otherwise be available cannot be utilised to meet the increasing demand for hospital beds which is likely to arise as National Insurance becomes consolidated, and the whole organisation settles down to steady work.

At the present time it is estimated that there are two and a half millions of insured persons within the metropolitan area. Of this two and a half millions of insured persons 100,000 every week will require medical attendance, of whom at least 5,000 must have, if their requirements are met, treatment as in-patients in a hospital. The whole of the voluntary hospitals in London do not provide many more than 11,000 beds, of which 10,000 in round numbers may be taken to be in hospitals which at present admit, as in-patients, insured persons suffering from severe maladies. Now these 10,000 beds are all beds that are available for hospital purposes, not only for residents in the metropolis, but for the members of their families and a number of cases of grave importance which are sent to the London hospitals by members of the medical profession and others from the home counties and elsewhere. The average stay of an in-patient in the best-managed London hospitals is about nineteen days, so that in due course, as matters stand at present, it is possible that there may be a demand for in-patient treatment by insured persons alone to the extent of 10,000 beds. If these figures prove to be approximately accurate it must become plain that, do what they will, the voluntary hospitals, unless very considerable extensions are made, cannot possibly undertake to provide sufficient accommodation for all insured persons resident within the metropolitan area who may require it week by week. The bare possibility of such an eventuality proves the prudence of the voluntary hospital managers, who are doing their best to help in every way the successful working of the National Insurance Act, 1911, in deciding to move cautiously, so that whilst they are willing, if it were possible, to admit every insured person who is suffering from serious illness or accident and requires in-patient treatment, they have not bound themselves in any way to continue this practice, except tentatively, until sufficient time has elapsed to test the requirements of the Act, and to see exactly what number of in-patient beds must be provided in London for the accommodation of insured persons alone.

In these circumstances the great need of the moment is for the Government to be alert to the position of affairs which actually prevails, not only in London but throughout the country, and by organisation and a careful consideration of all the existing means in the way of hospital beds which might be made available gradually to prepare the way for a consolidation of all existing agencies upon a basis which, whilst it preserves the voluntary system intact, will make available other beds in public hospitals, so as to guarantee that no insured person throughout the metropolitan area needing in-patient benefit shall fail to be able to obtain it immediately the requirement arises. The President of the Local Government Board, by the institution of a wise policy in conjunction with the London County Council, has been able already to make available 560 beds for cases of tuberculosis in the hospitals of the Metropolitan Asylums Board. The actual number estimated to be required for cases of tuberculosis in the metropolitan area is 1,000 beds, and steps are being taken, we understand, to bring the number of beds available in Metropolitan Asylums Board hospitals up to the required number.

### First Steps to Take.

The Poor-Law infirmaries, as we show in our article on the Fulham Infirmary on page 21 of the present issue, are so constructed and found as to be suitable, when freed from the taint of the Poor Law, and strengthened by a reduction in the number of beds existing in some of the larger wards and by an addition to the nursing staff, to take their place as public hospitals which could be readily utilised for the reception of insured persons needing such accommodation. It would be necessary also in most of the Poor-Law infirmaries, though not in all, to appoint a special medical staff of highly paid qualified men to do the surgical work and to act as consultants, as and where their services may be required. This step has already been taken in connection with some Poor-Law infirmaries, and it need not be difficult to extend the system to every public hospital should the plan here suggested be accepted as a basis for reorganising the whole of our present arrangements in regard to hospital treatment. It has always been a serious handicap for the poor that when the Poor-Law infirmary was first instituted the Act was "amended" (?) in the House of Lords by striking out the provision which threw these establishments open for clinical teaching. This is a point which must be provided for as a part of the reorganisation of the Poor-Law infirmaries and their conversion into public hospitals.

### Amendments to National Insurance Act, 1911.

We hope, therefore, that this great question will be thoroughly and promptly considered in all its bearings by Mr. John Burns and the Local Government Board, so that there may be neither delay nor difficulty, should occasion arise, to put at the disposal of the County Council for the benefit of its Insurance Committee the requisite number of extra beds for the in-patient treatment of insured persons, in addition to that supplied by the voluntary hospitals. If this had been done, and in the new Act now spoken of for amending the Insurance Act, 1911, powers were given to provide hospital benefit on the *pro rata* system we have consistently advocated in these columns, the whole of the existing buildings available for in-patients, whether in voluntary or public hospitals, could be readily brought into use, and so the most economical and efficient means for supplying the public needs in this respect could be happily secured by wise co-operation and administration. Any Act amending the National Insurance Act must further confer powers on the Local Government Board to make loans to the managers of voluntary hospitals, and to county and municipal councils which administer public hospitals, to enable new pavilions to be built in connection with both types of hospitals, in which accommodation could be provided for patients who would be paid for by Insurance Committees and public authorities on the *pro rata* basis, and for each class of the community, who would be greatly benefited by having placed at their disposal hospital accommodation which they might readily obtain in accordance with a scale of charges so arranged as to provide for the following:

(1) Those who, whether insured persons or not, could afford to pay the cost of their maintenance, plus 10 per cent. for medical service, during their hospital treatment and care.

(2) Patients who would pay a higher rate for better accommodation in the sense that it might be secured in a ward of two beds or of one bed, as the case might be, in accordance with the rate of payment per diem charged, plus 10 per cent. to pay the cost of medical treatment.

All loans made on this plan to any voluntary hospital or any municipal or county authority to be secured as a first charge on the buildings and patients' payments, the interest charged on such loans to be 3 per cent. per annum, plus 1 per cent. sinking fund, so that the amount advanced would be redeemed at the expiration of a fixed number of years. In this way, by using the national credit, without any permanent charge whatever to the tax or ratepayers, we might readily build up throughout Great Britain a system of hospitals by inter-communication and co-operation which would preserve the voluntary system intact and provide adequate hospital accommodation for all classes of his Majesty's lieges.

We have specially been dealing with the Poor-Law infirmaries in London because the circumstances in London, especially in reference to the powers and authority of the Local Government Board, differ from those which prevail in some other parts of the country, so that the exact position everywhere in regard to Poor-Law infirmaries must be ascertained and the necessary legislation, which in view of the precedent which London affords would be of a very simple character, would have to be enacted and would, we presume, form a portion of the proposed amending Act or Acts which must be passed in due course.

### Provision for Chronic Cases and the Old.

Assuming that the Poor-Law infirmary may be taken in hand and developed in some such directions as those we have ventured to indicate, should it be found that the demand for in-patient treatment by insured persons would absorb an unduly large number of beds in these establishments, then steps would have to be taken to provide for the large number of chronic cases and old persons, who require for the most part shelter and care rather than medical treatment and highly qualified nursing, so that these deserving poor people may not suffer hardship from the change, but rather benefit by it. There is, we are glad to say, evidence of a movement in agricultural districts for the establishment of old folks' homes in villages. The first of these is being organised by the Rev. A. H. Baverstock of Hinton Martel Rectory, Wimborne, who estimates that the welfare of the agricultural labourer and of the countryside in his district can be adequately secured so far as the old people and chronic invalids are concerned, by an expenditure of £1,300, of which £800 is required for buildings and the balance of £500 to constitute a small endowment. We should like to see the Hinton Martel proposal put into practical execution and submitted to every test, so as to demonstrate whether or not the idea is feasible. We should be glad to open our columns to a discussion on the points raised by this article, which are matters of the first interest.

